# Amplitude Reduction and Phase Shifts of Melatonin, Cortisol and Other Circadian Rhythms after a Gradual Advance of Sleep and Light Exposure in Humans

**DOI:** 10.1371/journal.pone.0030037

**Published:** 2012-02-17

**Authors:** Derk-Jan Dijk, Jeanne F. Duffy, Edward J. Silva, Theresa L. Shanahan, Diane B. Boivin, Charles A. Czeisler

**Affiliations:** 1 Division of Sleep Medicine, Harvard Medical School, Boston, Massachusetts, United States of America; 2 Division of Sleep Medicine, Brigham and Women's Hospital, Boston, Massachusetts, United States of America; Vanderbilt University, United States of America

## Abstract

**Background:**

The phase and amplitude of rhythms in physiology and behavior are generated by circadian oscillators and entrained to the 24-h day by exposure to the light-dark cycle and feedback from the sleep-wake cycle. The extent to which the phase and amplitude of multiple rhythms are similarly affected during altered timing of light exposure and the sleep-wake cycle has not been fully characterized.

**Methodology/Principal Findings:**

We assessed the phase and amplitude of the rhythms of melatonin, core body temperature, cortisol, alertness, performance and sleep after a perturbation of entrainment by a gradual advance of the sleep-wake schedule (10 h in 5 days) and associated light-dark cycle in 14 healthy men. The light-dark cycle consisted either of moderate intensity ‘room’ light (∼90–150 lux) or moderate light supplemented with bright light (∼10,000 lux) for 5 to 8 hours following sleep. After the advance of the sleep-wake schedule in moderate light, no significant advance of the melatonin rhythm was observed whereas, after bright light supplementation the phase advance was 8.1 h (SEM 0.7 h). Individual differences in phase shifts correlated across variables. The amplitude of the melatonin rhythm assessed under constant conditions was reduced after moderate light by 54% (17–94%) and after bright light by 52% (range 12–84%), as compared to the amplitude at baseline in the presence of a sleep-wake cycle. Individual differences in amplitude reduction of the melatonin rhythm correlated with the amplitude of body temperature, cortisol and alertness.

**Conclusions/Significance:**

Alterations in the timing of the sleep-wake cycle and associated bright or moderate light exposure can lead to changes in phase and reduction of circadian amplitude which are consistent across multiple variables but differ between individuals. These data have implications for our understanding of circadian organization and the negative health outcomes associated with shift-work, jet-lag and exposure to artificial light.

## Introduction

In humans, circadian rhythms in a variety of physiological and behavioral variables including core body temperature, urine production, hormones subjective alertness, cognitive performance, short-term memory, sleep propensity and sleep structure have been described (reviewed in [1], [2]). Under normal conditions, these rhythms are synchronized to the 24-h solar day and to each other, and exhibit specific phase relationships with the light-dark/activity-rest cycle, and with each other. For instance, plasma melatonin crests in the middle of the habitual dark/sleep episode, approximately two hours before the nadir of the core body temperature rhythm and approximately 4–6 hours before the crest of the cortisol rhythm. Many of these rhythms persist during sustained wakefulness under constant environmental and behavioral conditions. According to current understanding of circadian organization in mammals, all these rhythms are driven by a central circadian pacemaker located in the suprachiasmatic nucleus (SCN) of the hypothalamus, which coordinates multiple circadian oscillators in the periphery [3]. Single SCN neurons are competent circadian oscillators [4], and some of the genes involved in the generation of circadian rhythmicity have been identified [3].

The SCN receives light input from both classical photoreceptors (rods and cones), as well as from a more recently identified subset of intrinsically-photosensitive retinal ganglion cells [5], [6]. The phase (timing) of these endogenous circadian rhythms can be changed by an acute shift of the light-dark cycle [7]. The largest phase advances (shifts to an earlier time) are observed when light is administered in the late subjective night/early subjective morning, shortly after the core body temperature nadir, which in a young man sleeping from 00:00–08:00 would be approximately 06:00 h. Although in some prior experiments phase shifts were assessed for multiple variables and found to be equivalent [8], [9] few experiments have investigated whether individual differences in phase shifts are similar for a multitude of variables.

Circadian phase resetting by light is in general not thought to be accompanied by a change in the amplitude of circadian rhythms, and the negative consequences of circadian desynchrony are typically discussed within the framework of circadian phase rather than amplitude. Circadian amplitude reduction has, however, been observed when light is carefully timed to drive the oscillator to its singularity [10]–[12] but those findings and their interpretation remain controversial [13]. Whether individual differences in amplitude reduction are similar across endocrine and behavioral variables is currently not known. Although phase-shifting of the human circadian system was once thought to require bright light, dose-response studies indicate that the pacemaker is sensitive to moderate light, i.e. intensities typically observed in artificially lit living rooms or offices [14], both in the phase advance [15] and phase delay [16] regions of the phase response curve to light. A similar sensitivity is observed for the acute suppression of melatonin by light [14], [16] and the acute alerting effect of light [17]. Together, these studies indicate that the drive onto the pacemaker exerted by moderate light (∼100–150 lux) appears to be nearly half of the drive exerted by much brighter light (∼10,000 lux) [16]. The effect of a light stimulus is, however, also dependent on the prior history of light exposure [18]–[20], and to what extent moderate intensity light can affect multiple circadian rhythms remains unclear.

There is evidence to suggest that in addition to ocular light exposure, nonphotic stimuli, such as an imposed rest-activity cycle, also affect the phase of human circadian rhythms of melatonin, body temperature and other variables [21], [22]. Furthermore, it is well established that the rest-activity cycle and associated light-dark cycle also contribute to the observed amplitude of many variables, such as core body temperature, alertness and many endocrine variables [1].

The recent insights into the sensitivity of the human circadian pacemaker to photic and non-photic stimuli, and the emerging complexity of circadian organization led us to re-analyze phase resetting and amplitude effects of the sleep-wake and light-dark cycles on multiple endocrine, physiologic and behavioral variables. In particular, we aimed to investigate to what extent altered timing of the sleep-wake cycle and associated room and bright light cycles could affect the phase and amplitude of multiple circadian rhythms, and how changes in those rhythms correlated within individuals and differed between individuals.

## Materials and Methods

### Objectives

To investigate the extent to which the sleep-wake cycle and associated moderate and bright light exposure can affect the phase and amplitude of multiple circadian rhythms, and the extent to which individual differences in changes in phase and amplitude are correlated across variables. We selected a protocol in which the sleep-wake and light-dark cycle were advanced gradually to accomplish a 10-h phase-advance over a five day interval. This protocol is different from the most commonly used protocols in which the organization of the human circadian system is investigated by an acute shift of the sleep-wake cycle and the associated light-dark cycle. The protocol is also different from those few studies in which a gradually advancing sleep-wake schedule was used, because in those studies the daily advance was either shorter (30 min [23] or 60 min [24]) or imposed for only 3 days [25].

### Participants and screening

Data were obtained from 14 male participants [25.8 (2.7) (SEM) years], who were free of sleep complaints as assessed by a sleep disorders questionnaire, and in good physical and mental health. Extreme chronotypes [26] were excluded.

### Ethics

The experimental procedures and the procedure for obtaining informed consent were approved by the Brigham and Women's Hospital Human Research Committee, and the study was conducted in accordance with the principles outlined in the Declaration of Helsinki. Before participation, every subject received a description of the procedure and general purpose of the experiment and provided written informed consent.

### Protocol

**Figure 1:**
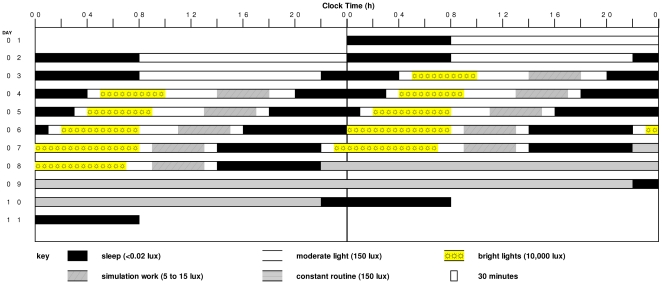
Raster plot of the experimental protocol. During the two baseline days sleep episodes were scheduled from 00:00 to 08:00 Thereafter, the sleep-wake schedule was advanced gradually resulting in a 10 hour advance of the sleep-wake schedule. Sleep episodes on day seven and eight were scheduled from 14:00 to 22.00. During scheduled sleep episodes, participants were exposed to darkness (<0.02 lux). During waking hours the participants were exposed to ∼90–150 lux moderate light. During the bright light treatment episodes, the moderate light participants remained in moderate light, whereas the bright light treatment participants received ∼10,000 lux of light. Both moderate light and bright light treated participants participated in dim light work simulations.

The study used a parallel group design, with seven participants in each group (moderate light group and bright light group), with the experimental protocol as shown in Fig. 1. Following two baseline days with 8-h sleep episodes scheduled from 00:00 to 08:00 h, the sleep-wake schedule was advanced such that after 5 days the sleep episodes were scheduled from 14:00 to 22:00. The 10-h advance was achieved gradually by scheduling the sleep episodes (SP) as follows: SP 3 (duration 6 h): 22:00 to 04:00 h; SP 4 (duration 7 h): 20:00 to 03:00 h; SP 5 (duration 7 h): 18:00 to 01:00 h; SP 6 (duration 8 h): 16:00 to 00:00 h; SP 7 and 8 (duration 8 h): 14:00 to 22:00 h. During scheduled sleep episodes, participants were in the dark (<0.03 lux) and were required to remain supine in bed. During waking hours the participants were exposed to moderate light (∼100–150 lux), with interventional exceptions as noted below.

Participants in the bright light group were exposed to bright indoor light (∼10,000 lux) for 5 to 8 hours each day on Days 4–8. The exact timing and duration of the overall daily light exposure was designed according to a mathematical model [27]–[29] to optimize the drive onto the circadian pacemaker. In this model, a low stiffness van Der Pol oscillator is influenced by light. The drive of light onto the pacemaker is I^1/3^ and maximum sensitivity to light occurs at the minimum of the core body temperature (CBT) rhythm, which on average occurs 1–2 hours before habitual wake time. In this model, light pulses centred after the CBT minimum will lead to phase advance shifts. The lighting schedule was designed primarily to impact the phase advance portion of the phase response curve on the five study days during which the bright light was presented, and to be robust against inter and intra-individual differences in circadian phase. To accomplish this, the duration of the bright light exposure was 5, 5, 6, 7.5 and 8 h in the early part of the waking episode on study days 4, 5, 6, 7 and 8 respectively. Bright light exposure was initiated 1 h after scheduled wake time on each of these days, with the exception of study day 7, when it was initiated 0.5 h after scheduled wake time. Thus, the timing and duration of the light pulses was such that even if considerable intra and inter individual differences in circadian phase were to occur, the center of the bright light pulse would likely be located after the CBTmin so as to induce a phase advance shift. Participants in the moderate light group remained in ∼100–150 lux of light during the hours that the bright light group were exposed to bright light. The protocol was designed to facilitate adaptation of astronauts during the pre-launch interval to a schedule required in preparation for a night launch of the space shuttle, as first implemented for STS-35 in 1990 [30]. Because astronauts had to participate in daily operational simulations during the pre-launch interval, all study participants were exposed to dim light conditions (∼5–15 lux) for four hours each day to simulate this training. The dim light exposure was scheduled so that the phase delay portion of the phase response curve received as little light as possible. The 4-h episode of dim light exposure started 6 h prior to SP 4, and 5 h prior to SP 5–8. After completion of the 10-h phase advance of the sleep-wake schedule, a 48-h constant routine was performed on study days 9–10 to assess the phase and amplitude of circadian rhythms.

### Exposure to bright light

During bright light exposure, light levels were increased to ∼10,000 lux. To minimize subject discomfort related to sudden changes in illuminance, bright light exposure episodes were preceded and followed by a 25-min episode during which time illuminance was gradually increased or reduced in 5 steps. The bright light was provided by ordinary wall-mounted fluorescent ‘cool white’ lamps. The fixtures covered the wall from floor to ceiling with 8-foot lamps arranged vertically. Light levels during bright light episodes were monitored and recorded every 10 minutes with a research photometer (International Light, Peabody, MA and Sper Scientific, Tempe, AZ). The detector was placed on the subject's forehead in the line of gaze. Both groups of participants wore clear, ultraviolet-filtering polycarbonate Ultra-Spec 2000 safety goggles with 4C coating (UVEX Winter Optical, Smithfield, RI) throughout bright light or room-light control exposures.

### Constant routine

The constant routine method was used to unmask the endogenous circadian component of the physiologic and behavioral rhythms [31]. During the constant routine, participants were restricted to a regimen of semi-recumbent posture, low activity, and continuous wakefulness in constant indoor lighting (90–150 lux). Wakefulness was enforced by a technician present in the room and verified by continuous polysomnographic recording. Daily nutritional and electrolyte needs were met by identical hourly snacks.

### Plasma melatonin, cortisol, and core body temperature collection

Blood samples were collected 1–3 times per hour via an indwelling forearm catheter connected to a 12-foot IV line (Liberty Medical LLC, Sterling, VA) during the baseline day (Study Day 2) and during the constant routine (Study Day 8–10). Samples were placed on ice and centrifuged within one hour of collection, and the plasma was frozen at −20°C. Hourly samples were assayed for plasma melatonin concentrations using a radioimmunoassay (Pharmasan Labs, Inc., Osceola, WI). Assay sensitivity was 7 pmol/L, intra-assay and inter-assay coefficients of variations were 8% and 15%, respectively. Blood samples obtained at 20 min intervals were assayed for plasma cortisol using a radioimmunosassay.

Core body temperature was collected at one minute intervals by a disposable thermistor (Measurement Specialites, Inc, Hampton, VA) inserted ∼10 cm into the rectum.

### Polysomnographic recordings

During all scheduled sleep episodes, electroencephalograms (C3-A2, C4-A1, O1-A2, O2-A1), submental electromyogram and electrocardiogram were recorded with a Nihon Kohden- 5208 or 4418 electroencephalograph. The signals were also digitized and stored on a Sun-386i work station on which the Nicolet-Ultra-Som sleep analysis was implemented (Nicolet Corp., Madison, WI). Polysomnograms of the 7th and 8th sleep episode were scored manually (DBB, DJD) in 30s epochs according to established criteria [32].

### Neurobehavioral performance assessment

Subjective alertness was measured three times per waking hour using a linear, nonnumeric, 100-mm bipolar (sleepy - alert) visual analog scale. Cognitive performance was assessed hourly by administering a calculation test consisting of 125 random pairs of two digit numbers. The subject was asked to add as many pairs as possible within an allotted 4-min test opportunity.

#### Analysis of plasma melatonin

The timing of the plasma melatonin rhythm at the beginning of the protocol and during the constant routine at the end of the protocol was assessed by: 1) the onset of plasma melatonin secretion (the clock-time when the plasma melatonin concentration rose above 43.1 pmol/L) [33]; 2) the time at which rising levels crossed the individual's 24-h mean value; 3) the time at which falling levels crossed the individual's 24-h mean value; and 4) the midpoint between the upward and downward mean crossing [34]. The duration of the secretory phase of the melatonin rhythm was defined as the width of the melatonin peak, i.e. the interval between the upward and downward mean crossing. The amplitude of the melatonin rhythm was assessed by computing the area under the curve (AUC) using the trapezoid method, and applying it to a standard segment of the melatonin curve, from 8 h before the midpoint until 8 hours after the midpoint of the melatonin rhythm. Baseline values, defined as the average plasma melatonin concentration in the interval 12-8 h before and 8–12 h after the melatonin midpoint, were subtracted from the data prior to computing AUC. The change of amplitude of the melatonin rhythm was assessed as the change in AUC between day 2 and day 9, with the value during day 2 set as 100% [100*((AUC Day9-AUC Day2)/AUC Day2)].

#### Analysis of core body temperature

In each subject the endogenous circadian phases and amplitude of the core body temperature data collected during the constant routine was determined by fitting a harmonic regression model in which it is assumed that the data can be described by a fundamental circadian sinusoid and its first harmonic as well as serially-correlated noise [35]. The first five hours and the final half hour of temperature data from the constant routine were excluded from analysis to eliminate masking effects of awakening from sleep and the masking associated with the preparation for the sleep episode following the constant routine.

#### Analysis of plasma cortisol

Phase and amplitude of individual cortisol profiles during the 24-h baseline period and the first 24-h period of the constant routine were assessed by indentifying the maximum and the minimum after the time series was smoothed by a 2-h running average. In addition, the phase, mesor and amplitude of plasma cortisol data, averaged per group, were estimated by fitting a sine wave and linear function to the data using PROC NLIN (SAS ® Institute Inc., Cary, NC, Version 6.12 or later).

#### Analysis of alertness and performance

Phase and amplitude of individual alertness and performance profiles during the constant routine were assessed by fitting the function Var = C+L*time+ A*sinus ([phase-time]/24.2) as implemented in PROC NLIN (SAS) to the data. In the function, C represents a constant, L the linear change in alertness or performance with time awake, A the amplitude of the circadian modulation of performance, and Phase the estimated maximum of the circadian modulation of alertness or performance.

#### Analysis of association between amplitude reduction in melatonin and the amplitude of other rhythms

To assess the association in changes in amplitude across variables we proceeded in two ways. First, in each individual, amplitudes of all variables were assessed separately and associations between those amplitudes were assessed using a correlation.

Second, participants (regardless of group) were divided into those who showed a suppression of melatonin greater than 50% and those who did not. Next, the average waveforms of other variables (core body temperature, cortisol, subjective alertness, performance) were computed for each melatonin suppression group. In this analysis, all variables were referenced relative to the timing of the core body temperature minimum observed during the CR.

### Analysis of sleep data

An estimate of the effect of the light exposure group on sleep was obtained by analyzing polysomnographically-assessed sleep parameters from the 7th and 8th sleep episodes, i.e., the last two sleep episodes before the constant routine when sleep timing was advanced by 10 h. In one subject, the data of sleep episode 8 could not be scored due to technical problems. Sleep latency data were log-transformed in order to be normally distributed prior to statistical tests.

### Statistical methods

SAS ® (SAS Institute Inc., Cary, NC, Version 6.12 or later) mixed model analyses of variance (PROC MIXED) and paired and unpaired two-tailed Student t-test were used as appropriate to assess the significance of phase and amplitude differences between groups. Factors in these analyses were Group (Moderate Light, Bright Light) and Day (Day 2, Day9) or Sleep Period (SP 7, SP 8), the latter factors being repeated.

The phase and 95% confidence intervals of the circadian rhythms of alertness and performance were estimated by fitting the data to a combined sine function and linear function using a nonlinear regression procedure (PROC NLIN). Associations between variables were assessed by Kendall Product Moment correlations (PROC CORR).

## Results

**Figure 2:**
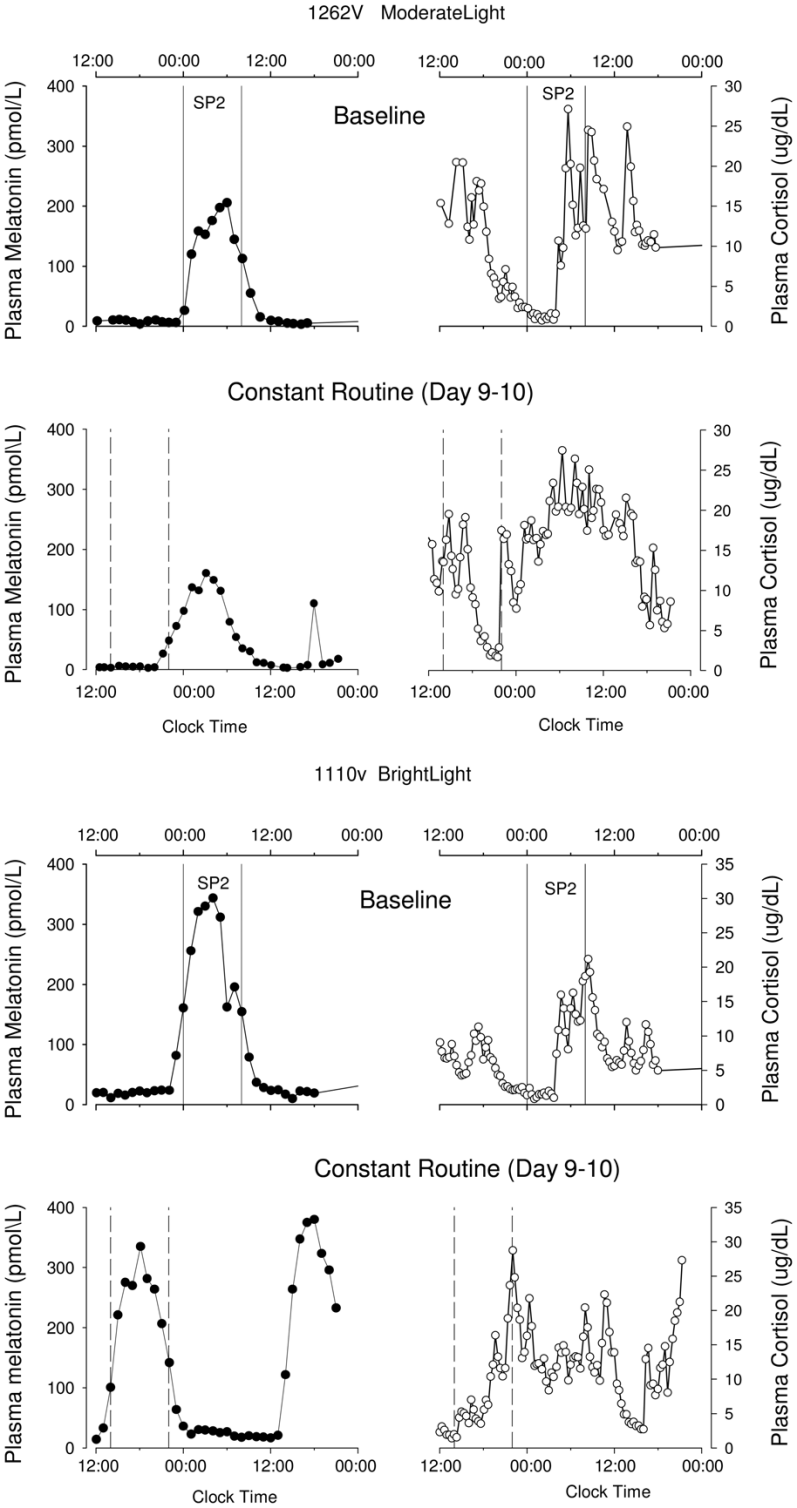
Plasma melatonin and cortisol rhythms. Plasma melatonin and cortisol rhythms before (Baseline – SP2 – upper panels) and after (Constant Routine – lower panels) the gradual shift of the sleep-wake schedule, in a participant treated with moderate light (1262V) and a participant treated with bright light (1110V). The vertical solid lines indicate the eight-hour scheduled sleep/darkness episode (SP2) on the baseline day (D2), the dotted vertical lines indicate the projected eight hour sleep episode (based on the sleep period of the preceding night) during the constant routine.

**Figure 3:**
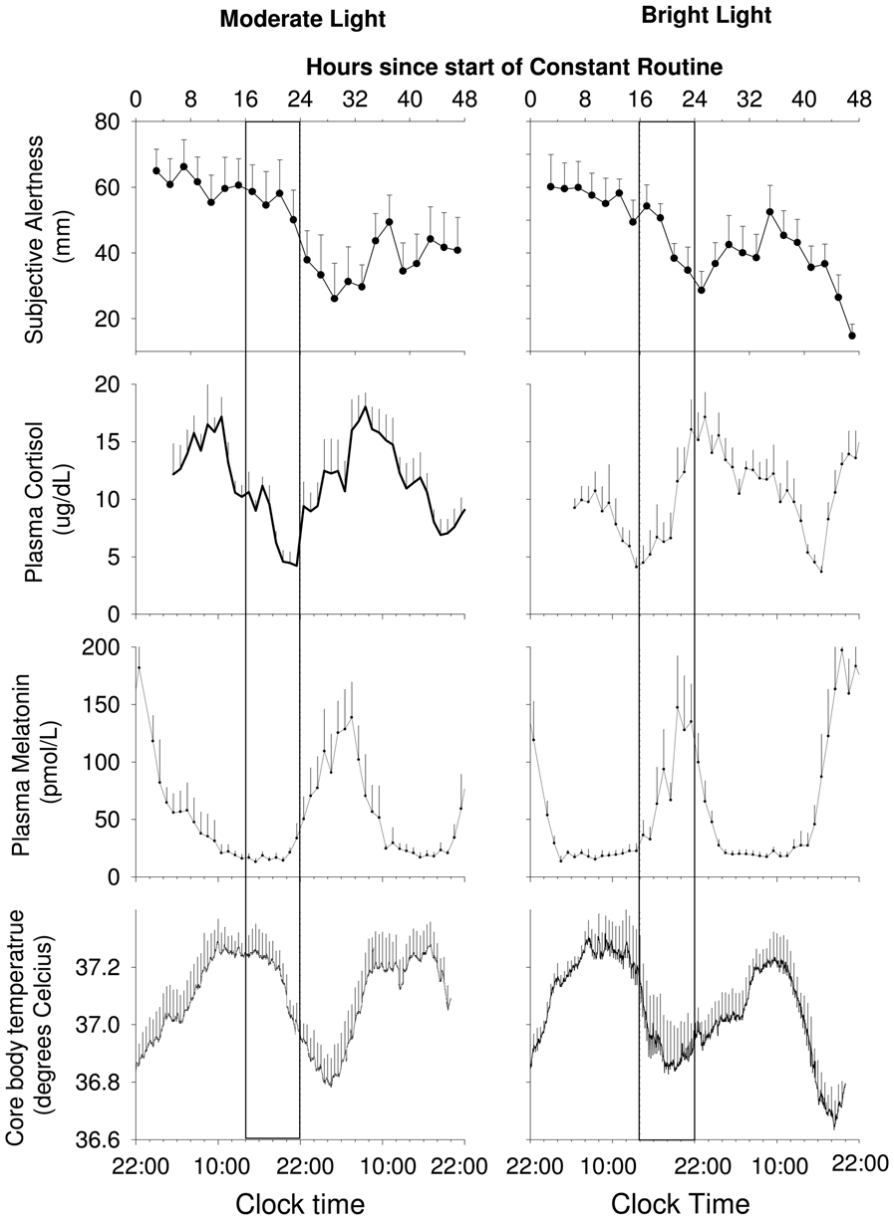
Average waveforms of circadian variables after moderate and bright light treatment. Time course of core body temperature, plasma melatonin, cortisol and subjective alertness during the constant routine in moderate light and bright light treated participants. Subjective alertness data were averaged per 2-h bins. All date are referenced to the projected wake time (22:00 clock-time). Box indicates the timing of the sleep episode on the previous day. Data represent means+/− SEMs.

Effects of treatment group on circadian phase were analyzed by assessment of the timing of individual phase markers (Table 1, Fig. 2) and by analyzing the average waveform of these variables for the two groups separately (Fig. 3).

**Table 1:** Timing of circadian phase markers.

	Moderate Light		Bright Light	
Phase marker	D2	CR	D2	CR
DLMO§§§	22:54 (7;0:27)	22:03 (5;0:53)	22:55 (7;0:14)	14:49 (7;0:47)###
Mel-Up§§§	23:32 (7;0:23)	22:43 (5;1:01)	23:48 (7;0:09)	14:39 (7;0:47)###
Mel-Down§§§	08:52 (7;0:28)	07:07 (5;0:57)	08:22 (7;0:13)	22:12 (7;0:58)###
Mel-Mid§§§	04:12 (7;0:23)	02:57 (5;0:59)	04:05 (7;0:09)	18:26 (7;0:52)###
Cortisol-Min§§§	01:05 (6;0:46)	22:49 (5;01:46)	00:36 (7;0:52)	14:25 (7;0:41)###
Cortisol-Max§§§	09:04 (6;0:34)	05:25 (5;02:36)	09:00 (7;0:41)	22:05 (7;0:19)###
Temp-Min§§	NA	03:43 (7;01:01)	NA	22:02 (7;01:15)
Alert-Min	NA	01:26 (7;01:41)	NA	22:17 (7;01:17)
Perfor-Min	NA	04:13 (7;01:18)	NA	0:08 (7;01:30)

Timing [h:min (n;SEM)] of Dim Light Melatonin Onset (DLMO), melatonin-upward, melatonin midpoint, and melatonin downward crossing, cortisol minimum, cortisol maximum, core-body temperature minimum, alertness and performance minimum at baseline (D2) and during the Constant Routine (CR) in the Moderate Light and Bright Light condition. Minima for Alertness and Performance are the minima of the circadian component.

### p<0.001: D2 vs. CR;

§§ : p<0.01;

§§§ :p<0.001 Moderate Light vs. Bright Light during CR.

P values were derived from two factor Mixed Model ANOVA for those variables for which estimates were available at Baseline and During the CR or Students t-test for those variables for which estimates were available during the CR only.

### Plasma melatonin phase

The mixed model ANOVA yielded a significant effect of Group and Day as well as a significant interaction between these factors for the melatonin phase assessed by DLMO, upward crossing, midpoint, and downward crossing (p<0.001 for all variables and all effects). Contrast analyses revealed that the timing of the plasma melatonin rhythm as assessed by these four phase markers was not significantly different (p>0.3 in all cases) between the two groups at the baseline assessment on Day 2–3, with a rise in plasma melatonin levels occurring prior to bed time, and peak levels during the scheduled sleep episode (see Table 1 and Fig. 2 and 3). On study Day 9 following the advance of the sleep schedule, the timing of the onset, upward mean crossing, midpoint, and downward mean crossing of the plasma melatonin rhythm was significantly different between the two groups (p<0.001 in all cases). In the bright light group, each of the phase markers of the plasma melatonin rhythm had advanced significantly compared to the baseline assessment (p<0.001 for all phase markers) by at least 8 h (see Table 1), such that it was rising prior to and peaking in the middle of the advanced sleep episode. In contrast, in the moderate light group the timing of the melatonin rhythm did not change significantly from the baseline assessment (p>0.3 in all cases; see Table 1). This resulted in the peak of melatonin secretion occurring on average after scheduled wake time in the moderate light group (see Fig. 3). Note that for two participants in the moderate light group (1025 and 1141) we were not able to assess phase of the melatonin rhythm after the intervention, because the amplitude of the melatonin rhythm was so small that the DLMO and other phase markers could not be assessed reliably. These two subjects showed robust amplitudes of their melatonin rhythm on Day Two. Information on the phase shifts for each participant is presented in Table S1.

### Plasma cortisol phase

The mixed model ANOVA yielded a significant effect of Group, a significant effect of Day, and a significant interaction between these factors for the timing of the cortisol rhythm as assessed from the maximum or minimum (p<0.02 in all cases). Contrast analyses demonstrated that the timing of the plasma cortisol rhythm was not significantly different between the two groups at the baseline assessment on Day 2–3 (p>0.3 in all cases), with the minimum of plasma cortisol levels occurring around bed time, and peak levels occurring at the beginning of the wake episode (see Table 1 and Fig. 2 and 3). On study Day 9 following the advance of the sleep schedule, the timing of the minimum and maximum of the plasma cortisol rhythm was significantly different between the two groups (p<0.001). In the bright light group, the minimum of the plasma cortisol rhythm had significantly advanced (p<0.001) by on average 10 hours, such that it remained coincident with the beginning of the scheduled sleep episode. In contrast, in the moderate light group the timing of minimum of the cortisol rhythm did not change significantly (p>0.2) from the baseline assessment (see Table 1) with an average advance of only 2 hours. Analysis of the average waveform (Fig. 3) revealed that at the end of the experiment in the bright light group the scheduled sleep episode coincide with the rising limb of the cortisol rhythm, similar to the baseline condition. In contrast, the maximum of the average waveform of cortisol in the moderate light group was located at 7:58 (95% CI 7:26–8:32), which was well before the beginning of the scheduled sleep episode. Thus in the moderate light group the scheduled sleep episode coincided with the declining limb of the cortisol rhythm (see Fig. 3).

### Core body temperature phase

On Day 9 after the gradual sleep/wake schedule inversion, the temperature minima measured during constant routines occurred significantly earlier in the bright light group than in the moderate light group (22:02±1:15 h vs. 03:43±1.01 h; p<0.005). The timing of the temperature rhythm was such that in the bright light group the nadir occurred near the end of the scheduled sleep episode, while in the moderate light group the temperature nadir occurred on average 5.5 h after the scheduled wake time (see Fig. 3).

### Alertness and performance phase

Alertness and Performance both exhibited a decline during the CR associated with the extended duration of wakefulness. Following ≥16 hours of wakefulness, alertness levels appeared to co-vary with the core-body temperature rhythm (illustrated for alertness in Fig. 3). After approximately 24 hours awake, subjective alertness co-varied with the core-body temperature cycle in both groups (see upper panels of Fig. 3), such that in the bright light group the first observed minimum was located close to the end of the scheduled sleep episode, whereas in the moderate light group it was located several hours later, after the beginning of the scheduled wake episode. Fitting the average alertness curves yielded an estimate of the timing of the minimum in the bright light group at 22:18 h (95% CI 20:58–23:39), just after scheduled wake time, whereas in the moderate light group the minimum of the average curve of alertness occurred at 03:32 h (95% CI 2:03–5:03), more than 5 hours after scheduled wake time. Alertness levels then improved, coincident with the rising limb of the core body temperature rhythm. Analysis of the calculation performance data showed that the minimum of the average curve of performance in the bright light group occurred at 00:59 h (95% CI 23:33–02:26), whereas it occurred three hours later in the moderate light group (03:59 h; 95% CI 02:53–05:06). This analysis at the group level was consistent with the analysis of the timing of the minima on an individual basis, although in that analysis only the difference in the timing of the minimum for performance was significantly different between the groups (Table 1).

### Individual differences in the timing of circadian rhythms after the intervention: association across variables

**Figure 4:**
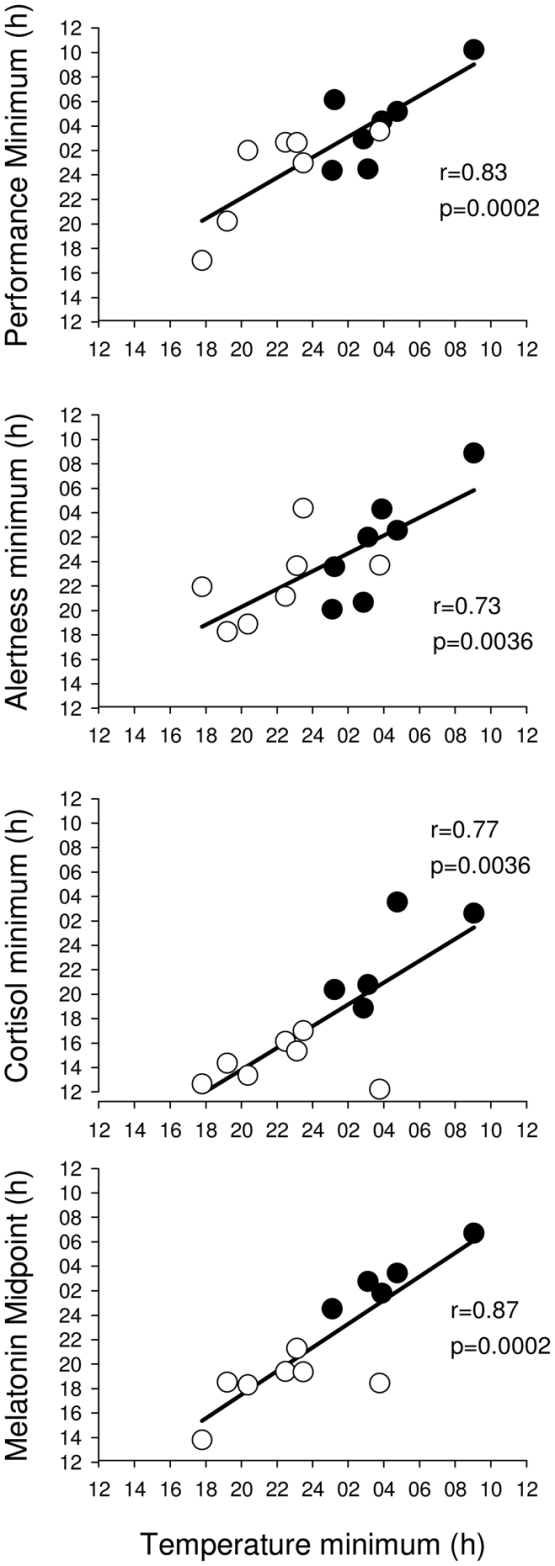
Association between the timing of circadian variables. Association between the **t**iming of melatonin midpoint, cortisol minimum, circadian minimum of alertness and performance and the timing of the temperature minimum during the constant routine in participants exposed to moderate light (filled symbols) or bright light (open symbols).

We examined the association between individual differences in the timing of the various variables during the CR, independent of the treatment group and found that they were highly correlated (Fig. 4). Thus, the correlation between the timing of the minimum of the core-body temperature and the cortisol minimum was 0.766 (n = 12; p = 0.004) whereas the correlation with the cortisol maximum was 0.605 (n = 12; p = 0.037). The correlation between the body temperature nadir and various indices of melatonin phase was 0.871 (p<0.001) for the timing of the melatonin midpoint, and 0.907 (p = <0.001) for the DLMO. The timing of the body temperature nadir was also correlated with the timing of the minimum of the circadian component of alertness (r = 0.730; p<0.005) as well as the minimum component of performance (r = 0.833; p<0.001).

### Melatonin amplitude

**Figure 5:**
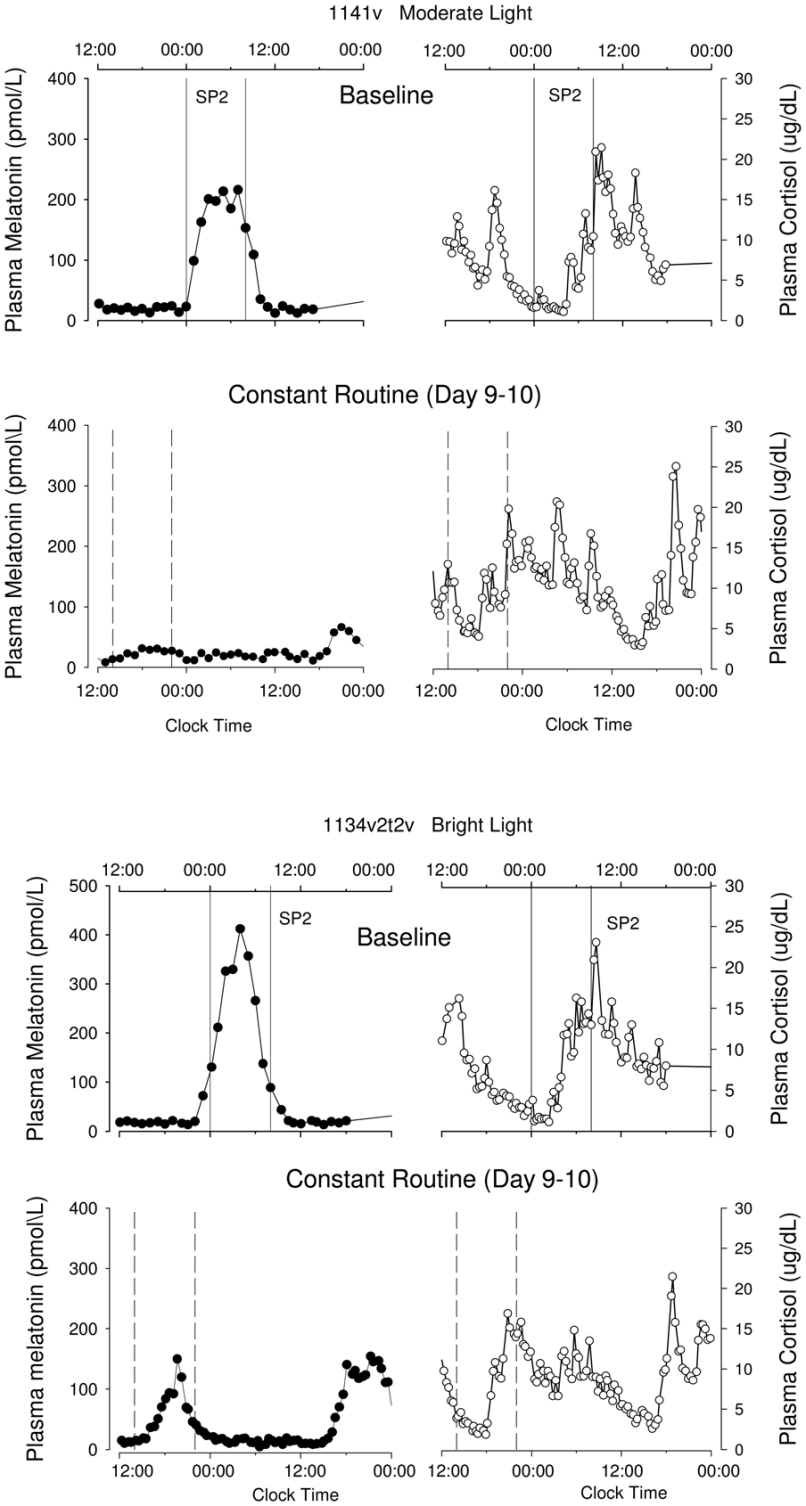
Examples of amplitude reduction. The plasma melatonin (filled symbols) and cortisol rhythm (open symbols) during the baseline day and SP2 and the constant routine (Day 9–10) in a bright light (1134) and moderate light (1141) treated subject. In both participants a reduction of the plasma melatonin amplitude as well as changes in the cortisol rhythm can be observed.

Visual inspection of the melatonin profiles revealed that in some subjects no discernible rhythm was present during the CR despite robust rhythmicity at baseline. These reductions were observed in both treatment groups (for two examples see Fig. 5; Table 2 for averages per treatment group).

**Table 2:** Amplitude measures of circadian markers.

	Moderate Light		Bright Light	
	D2	CR	D2	CR
Mel AUC	98.1 (7;6.7)	47.6 (7;13.1)##	93.7 (7;14.1)	45.8 (7;13.4)##
Mel Height	228.7 (7;11.4)	133.9 (7; 34.4)#	250.9 (7; 41.2)	142.8 (7; 34.9)##
Mel Width	9:19 (7;0:24)	8:11 (5; 0:26)	8:35 (7; 0:12)	7:34 (7; 0:21)##
Cort AUC	215.7 (6;15.65)	262.4(5; 25.32)#	221.7 (7; 23.8)	244.1(7; 30.4)
Cort Min	1.54 (6; 0.18)	3.94 (5; 0.76)##	2.13 (7; 0.47)	2.61 (7; 0.38)
Cort Max	18.22 (6; 1.72)	18.70 (5; 1.75)	18.74 (7; 1.83)	19.53 (7; 2.17)
Cort Range	16.68 (6; 1.72)	14.76 (5; 2.07)	16.61 (7; 1.41)	16.92 (7; 2.0)
Temp Amp	NA	0.27 (7;0.03)	NA	0.30 (7;0.02)
Alert Amp	NA	10.85 (7; 3.31)	NA	9.49 (7; 2.11)
Perf Amp	NA	8.83 (7;2.8)	NA	6.51 (7;1.0)

Amplitude measures for melatonin (Mel), cortisol (Cort), core body temperature (Temp), alertness (Alert) and performance (Perf) on D2 and the CR separately for the moderate light and bright light treatment group. Melatonin height: pmol/L; Melatonin area under the curve (AUC) nmol/L*min; Melatonin width: h:min. Cortisol measures are in µg/dL, temperature measures in degrees Celsius, Alertness in mm and Performance in number of additions attempted.

## p<0.01 CR vs. D2 per treatment group (see text for overall statistical significance for effect of Day (i.e. CR vs. D2). For core body temperature, alertness and performance data were not available (NA) during D2.

Mixed model ANOVA yielded a significant effect of Day for the height of the melatonin rhythm (p<0.004), the area under the curve (P<0.001), the average melatonin concentration (p<0.0003), as well as the width of the melatonin rhythm (p<0.01) with no significant effect of Group (p>0.09 in all cases) and no significant interaction between these two factors (p>0.5 in all cases). The average reduction of the area under the curve (CR-D2 expressed as percentage of the value at D2) was 53.6% (n = 7; SEM = 12.1%) in the moderate light and 51.7% (n = 7; SEM = 10.5%) in the bright light group. For the height of the melatonin rhythm the reductions were 43.6% (7; 13.8) and 44.0% (7; 9.5) for the moderate light and bright light condition respectively. For the duration (width) of the melatonin secretion phase the reductions were 12.9% (5; 7.6) and 12.0% (7; 2.7) for the moderate light and bright light condition respectively. Thus, several amplitude measures of the melatonin rhythm as assessed under constant-routine conditions following a gradual advance of the sleep-wake cycle were significantly reduced compared to the amplitude assessed at baseline in the presence of a sleep-wake cycle. Information on the change in melatonin amplitude for each participant is presented in Table S1.

### Cortisol amplitude

Several amplitude measures of the cortisol rhythm were also affected by the protocol (Table 2). A significant effect of Day was observed for the minimum of the cortisol rhythm (p = 0.01), with no significant effect of condition (p = 0.45) or interaction (p = 0.063). The minimum of the cortisol rhythm was significantly higher on Day 9 compared to Day 2 (p = 0.01) with an average increase (CR-D2 as % of D2) of 165.8% (5;26.8) in the moderate light and 54.7% (7;39.5) in the bright light group.

The area under the curve was significantly greater (p = 0.039) on Day 9 compared with Day 2. No effect of Group (p = 0.78) or interaction (p = 0.36) was observed for this measure and the average increase was 25.6% (5;6.1) and 11.4% (7;10.1) in the moderate light and bright light group respectively.

### Core body temperature, alertness and performance amplitude

For core body temperature, alertness and performance, circadian amplitude could only be assessed during the Constant Routine (Table 2). No differences between groups were observed for any of these variables, and in both groups a wide range of amplitude values were observed across individuals.

### Association between melatonin amplitude reduction and amplitude of other rhythms

The amplitude reduction as measured by area under the melatonin curve varied considerably between subjects in each group. In the bright light group it varied from 12.3–84.3% whereas in the moderate light group the range was 16.6–93.7%.

**Figure 6:**
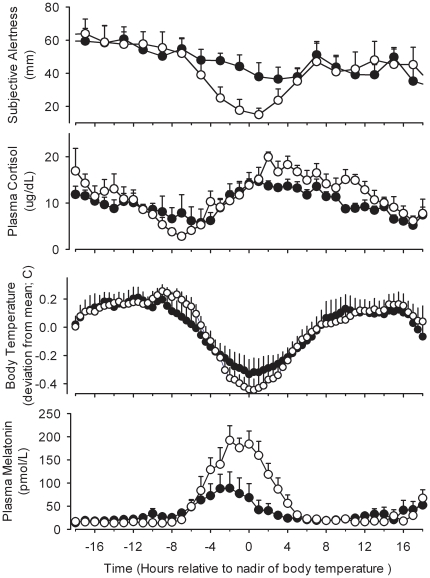
Average waveforms of circadian variables and amplitude reduction. Average waveform of subjective alertness, plasma cortisol, core body temperature and plasma melatonin during a constant routine in participants with a reduction in melatonin amplitude >50% (closed symbols; n = 7) compared to those in whom the reduction was <50% (open symbols; n = 7). All data are aligned with the timing of the fitted minimum of the core body temperature rhythm. Error bars indicate 1 SEM.

**Figure 7:**
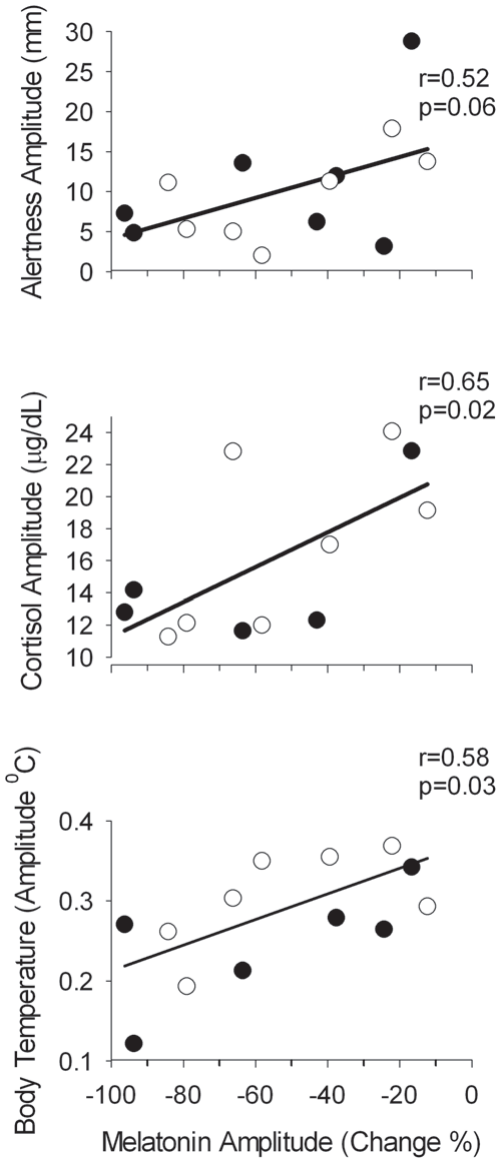
Association between the amplitude of circadian variables. Association between the amplitude of body temperature, cortisol, and alertness during the constant routine and the reduction in the amplitude of melatonin in participants exposed to moderate light (filled symbols) or bright light (open symbols).

To investigate whether reduction of melatonin amplitude was associated with changes in the amplitude of other rhythms we proceeded in two ways. We first divided subjects into those with greater (>50%, n = 7) or less (<50%, n = 7) reduction in melatonin amplitude between Day 2 and Day 9 and compared the average waveforms of melatonin, core body temperature, cortisol, and alertness between these two groups (Fig. 6). We then assessed the correlations between the amplitude measures of these variables at an individual level (Fig. 7). In the stronger suppressors, the amplitude reduction was primarily related to a reduction of the maximum of melatonin secretion and not due to an increase in the baseline (see lower panel of Fig. 6). As a group, the strong suppressors showed a significantly lower core body temperature amplitude, demonstrated by a lower fitted mesor of the average core body temperature curve (Stronger suppressors: 37.03°C; 95%CI: 37.02–37.05; vs. Weaker suppressors: 37.09; 95%CI:37.07–37.11) and a lower fitted core body temperature amplitude (Stronger suppressors: 0.23°C; 95%CI:0.22–0.25; Weaker suppressors: 0.29; 95%CI:0.27–0.31; Fig. 6). A significant correlation between melatonin amplitude reduction and the amplitude of the core body temperature rhythm was observed (n = 14, r = 0.58;p = 0.028; Fig. 7).

The cortisol amplitude also differed between the stronger and weaker melatonin suppressors. In the stronger suppressors, the fitted mesor of the plasma cortisol rhythm was significantly lower than in the weaker suppressors (Stronger Suppressors: 10.15 ug/dL, 95%CI: 9.70–10.60; Weaker Suppressors: 11.83 ug/dL, 95% CI: 11.28–12.37), and the amplitude of the cortisol rhythm was also significantly lower (Stronger Suppressors: 3.35 ug/dL, 95%CI: 2.73–3.97; Weaker Suppressors: 5.54 ug/dL; 95% CI: 4.80–6.29; Fig. 6). The correlation between the change in amplitude of cortisol, defined as the difference between minimum and maximum, and the change in amplitude in melatonin was 0.57 (n = 12; p = 0.051; Fig. 7).

Suppression of the plasma melatonin rhythm also affected the time course of performance and alertness, such that the stronger suppressors showed a near linear decline in alertness with time awake, while the weaker suppressors showed a marked deterioration of alertness coinciding with the peak of the melatonin rhythm, in addition to the linear trend (see upper panel of Fig. 6). The overall amplitude of the alertness rhythm was significantly lower in the stronger suppressors (Stronger Suppressors: 4.59 mm, 95% CI: 1.27–7.90; Weaker Suppressors: 16.93 mm, 95% CI: 11.60–22.27). Overall the stronger suppressors were more alert throughout the extended wake episode on Day 9, and their average waveform of alertness had a mesor of 46.25 mm (95%CI 43.93–48.56) vs. 39.88 mm (95%CI 36.17–43.59) in the weak suppressors. The correlation between the change in amplitude of melatonin and the amplitude of alertness was 0.52 (n = 14;p = 0.058; Fig. 7). The fitted amplitude of the performance rhythm was also lower for the stronger suppressors (stronger suppressors: 6.7 calculations attempted, 95%CI 3.5–9.8; weaker suppressors: 10.4 calculations attempted, 95%CI 6.4–14.5, See Fig S1). The correlation of the estimates of amplitude for this measure did however not correlate significantly with melatonin suppression.

It should be noted that a reduction in the amplitude of the alertness rhythm resulted in higher alertness during the biological night in the stronger melatonin suppressors.

### Comparison of sleep in moderate light and bright light groups

Sleep parameters averaged across sleep periods 7 (SP-7) and 8 (SP-8) are presented in Table 3. Mixed model ANOVA with factors Group and Sleep-Period revealed a significant effect of Group on sleep latency (p<0.04) with no significant effect of Sleep-period or interaction between these factors. The average sleep latency on Nights 7 and 8 (after the sleep episode had been advanced by 10 h) was less than 5 minutes in the moderate light group, significantly shorter than sleep latency in the bright light group (Table 3).

**Table 3:** Polysomnographically assessed sleep parameters in the Moderate Light and Bright light group.

	Moderate Light	Bright Light	Moderate vs. Bright
Sleep Latency (min)	4.6 (1.6)	18.4 (9.8)	P = 0.04
REMS Latency (min)	49.2 (9.4)	64.5 (5.3)	NS
Total Sleep Time (min)	348.4 (12.6)	380.6 (19.7)	NS
Wake After Sleep Onset (min)	128.0 (12.7)	81.3 (19.0)	NS
Stage 1 (min)	33.5 (3.2)	39.8 (2.9)	NS
Stage 2 (min)	147.1(8.6)	187.8 (12.2)	P = 0.02
Stage 3 (min)	32.0 (4.4)	37.9 (6.1)	NS
Stage 4 (min)	70.2 (11.6)	31.2 (13.5)	P = 0.05
Slow Wave Sleep (min)	102.1 (11.7)	69.1 (17.9)	NS
REM Sleep (min)	65.6 (4.5)	83.9 (8.8)	NS
Movement Time (min)	2.8 (1.0)	3.4 (1.2)	NS
Stage 1%	9.9 (0.9)	10.9 (0.8)	NS
Stage 2%	42.2 (2.4)	49.8 (3.1)	NS
Slow Wave Sleep %	29.4 (2.9)	17.8 (3.9)	P = 0.4
REM Sleep %	18.6 (1.0)	21.6 (1.9)	NS

Mean and SEMS for sleep parameters for the two treatment groups. Data represent the averages of sleep episodes 7 and 8 for all subjects, with the exception of subject 1041 from the Bright Light group in whom only sleep episode 7 was included. n = 7 in each condition. % represents the percentage of that sleep stage expressed relative to total sleep time. P values indicate the significance of the difference between the Treatments as assessed in a two-factor Mixed Model ANOVA with factors Treatment and Sleep period. Sleep latency data were log-transformed prior to statistical tests.

No significant difference in total sleep time was observed between the two groups, but wake after sleep onset (WASO) tended to be lower in the bright light group (p<0.07). This reduction of WASO in the bright light group was accompanied by a significant increase in the duration of stage 2 which was particularly pronounced during SP-8 (stage 2 during SP-8 Bright Light vs. Moderate Light: p = 0.012). No significant difference in the duration of SWS was observed, but the duration of stage 4 was shorter and the percentage of SWS was lower in the bright light group than in the moderate light group and in particularly so during SP-8 (SWS% during SP-8 Bright Light vs. Moderate Light: p = 0.014).

### Comparison of sleep in strong and weak melatonin suppressors

Sleep parameters were compared in subjects in whom melatonin amplitude was reduced by more than 50% to those in whom it was less reduced. A mixed model ANOVA with factors Melatonin-suppression and Sleep-period did not yield a significant effect for the factor Melatonin-suppression for any of the sleep parameters. However, the interaction between the factor Melatonin-suppression and Sleep-period was significant for TST (p<0.05), Stage Three (p<0.03), Stage Four (p<0.01) and SWS (p<0.01). This interaction primarily represented a significant increase of TST (LSMEANS estimate: 95.8 min, SEM 31.4, P<0.01) from sleep period 7 to 8 in the group in which melatonin was only moderately suppressed and a reduction of Stage Three (14.8 min, SEM: 6.8; P = 0.05), Stage Four (25.4 min, SEM: 7.8; P<0.01) and SWS (40.1 min, SEM:11.8; P<0.01) from sleep period 7 to 8 in the group in which melatonin was strongly suppressed.

## Discussion

### Contribution of bright light and moderate light to maintaining synchrony of endogenous circadian rhythms with advanced sleep-wake cycle

The present data show that when the sleep-wake cycle is gradually advanced over several days and the associated light-dark cycle consists of only moderate light, multiple circadian rhythms desynchronize from the sleep-wake and light-dark cycle. This indicates that on average the drive of moderate light and the sleep-wake cycle onto the circadian pacemaker is insufficient to induce a phase advance of 2 h per day. By contrast, exposure to bright light for several hours following the sleep episodes facilitated the maintenance of the normal phase relationship between multiple circadian rhythms and the shifted sleep-wake cycle. The pattern of bright light exposure was designed to cause daily phase advance shifts sufficient to overcome the 0.2 h daily phase delay that would be expected based on the 24.2-h average period of the human circadian system [36] and the additional 2 h daily shift related to the advancing sleep-wake cycle. This bright light exposure schedule led to a phase advance of the melatonin rhythm in all participants, although individual differences were observed. In comparison, in the moderate light group, on average no significant phase-advance shift of the melatonin rhythm was observed, and some individual differences were present. Importantly, no phase delays were present in the moderate light treatment group indicating that the drive of the sleep-wake cycle and moderate light onto the circadian pacemaker was sufficient to counteract the phase delay associated with an average intrinsic period of 24.2 h, in accordance with studies showing that dim light is sufficient to maintain entrainment to the 24-h cycle [37]. Other studies using a progressively advancing schedule have reported effects of exposure to moderate light and shifted sleep-wake cycles on circadian rhythmicity [23], [24], [38] and taken together these data indicate that moderate light and feedback from the sleep-wake cycle exert a small drive onto the pacemaker, however that drive was insufficient to advance the circadian system by 2 h per day in our study.

### Individual differences in timing of circadian rhythms after bright and moderate light exposure: changes in circadian phase are similar for multiple variables

The assessment of the endogenous circadian phase of multiple rhythms under constant routine conditions at the end of the protocol indicated that individual differences in the timing of the rhythms were highly correlated across variables. This implies that under both light conditions the phase shifts of body temperature, plasma melatonin, plasma cortisol and alertness were of similar magnitude. This corroborates previous findings [39] [8] [25], [40] and indicates that all these rhythms are driven by the same light-sensitive pacemaker, presumably the SCN. This simultaneous change in the timing of multiple variables is likely to be mediated by the identified pathways from the SCN to the effector organs, i.e. pineal and adrenal cortex have been described, although the mechanisms by which the SCN regulates core body temperature alertness and performance is less clear.

### Reduction in circadian amplitude: correlation across variables

All amplitude measures of the melatonin rhythm indicated a reduction in circadian amplitude during the constant routine following the sleep-wake schedule inversion compared with the amplitude at baseline, i.e. in the presence of a sleep-wake and dark-light cycle. The reduction in amplitude primarily reflected a reduction in the maximum values of melatonin rather than an elevation in minimum values. On average, the change in melatonin amplitude did not differ between the bright light and moderate light groups and the large range of inter individual differences in range of amplitude changes was similar in the two groups. Amplitude reduction in melatonin was associated with lower amplitude of body temperature and cortisol. Whereas for body temperature the amplitude reduction represented elevated minima and reduced maxima, the change in cortisol amplitude appeared more related to elevated minima, although a reduction in maximum values cannot be excluded.

### Circadian desynchrony and amplitude reduction: effects on alertness, performance and sleep

Sleep and alertness and performance during wakefulness are determined by an interaction of circadian rhythmicity and sleep-wake homeostasis, such that consolidation of sleep and wakefulness depends on an appropriate phase relationship between the sleep-wake cycle and endogenous circadian rhythmicity [41]. According to this concept, a wake-promoting signal which becomes progressively stronger during the biological day opposes the decline in alertness and performance associated with sustained wakefulness. At the end of the biological day, this wake promoting signal dissipates and under normal conditions sleep will be initiated. However, if wakefulness is sustained beyond a typical ∼16 h wake episode, as was the case in our protocol or during night shift work, alertness and performance will deteriorate. The present data show that appropriate timing of the maximum of alertness and performance relative to the sleep-wake cycle correlates with the appropriate timing of other circadian rhythms such as melatonin. In the initial part of the constant routine, no differences between the two groups were observed but when the duration of prior wakefulness increased, differences between the two groups emerged. During the projected sleep episode, the light-treated group exhibited the adaptive decline of alertness with a minimum shortly after the minimum of the core body temperature rhythm, whereas in the control group alertness, and to a lesser extent performance, remained high in the initial part of the projected sleep episode and did not reach a minimum until 8–10 hours into the scheduled wake-episode. This time-course is consistent with previous reports of the interaction of circadian and wake dependent determinants of alertness and performance [42] [43] and with the observed phase differences between the two groups for other variables like melatonin, body temperature and sleep propensity.

The current data also show that when the amplitude of circadian rhythms is reduced, alertness and performance decline in a near linear manner during sustained wakefulness, i.e. the homeostatic component dominates the time course. Interestingly, this reduced circadian amplitude leads to higher levels of alertness and performance during the circadian night. The implication from this is that a reduction of circadian amplitude may facilitate the ability to stay awake at night, and that being better-able to stay awake at night may be indicative of a reduced circadian amplitude.

Analysis of sleep propensity and structure by polysomnography corroborated the interpretation that bright light treatment led to a more appropriate phase relationship between the scheduled sleep-wake cycle and endogenous circadian rhythmicity. In the moderate light group, the scheduled sleep episode was located before the onset of melatonin secretion and coincided with the plateau of the core body temperature rhythm. In contrast, in the bright light treatment group the scheduled sleep episode coincided with the phase of melatonin secretion and the falling limb on the core body temperature rhythm, as is usual during entrainment [2]. This difference in the phase relationship between the circadian system and scheduled sleep was associated with differences between the two groups on a number of sleep measures. Wake after sleep onset was lower in the bright light-treated group compared with the moderate light group. This finding is in accordance with observations from forced desynchrony experiments [44] [45] [46], which demonstrated that sleep consolidation in young adults can be maintained for 8 hours when sleep is initiated just after the onset of melatonin secretion, on the falling limb of the core body temperature rhythm.

The observation that sleep latencies were longer in the bright light group may at first glance seem to be at odds with the notion that the near normal phase relationship between sleep and circadian rhythmicity in this treatment group improves sleep. A variety of protocols have demonstrated that the circadian rhythm of sleep propensity reaches its nadir during the latter part of the habitual waking day, such that shortly before habitual bedtime, i.e. just before the onset of melatonin secretion the circadian drive for wakefulness is strongest [44], [47] When we are normally entrained, this circadian drive for wakefulness near the end of the habitual waking day results in a “wake maintenance zone” [48] or “forbidden zone for sleep” [49], which not only allows us to remain awake despite having been awake for many hours, but makes it difficult to fall asleep quickly. Thus, participants in the light treatment group, who exhibited a normal phase of entrainment at the end of the protocol, showed normal sleep latencies. In contrast, the moderate light participants showed significantly shorter sleep latencies. Because the timing of their circadian rhythms had not changed significantly while the timing of their sleep had advanced by 10 hours, they were initiating sleep in the ‘circadian afternoon’, a circadian phase at which the drive for waking is only moderate. This, together with cumulative sleep disruption on the prior advanced sleep episodes, likely contributed to the very short sleep latencies in the moderate light group. Although no major effects of moderate and bright light treatment or amplitude reduction on sleep structure were observed, some effects were puzzling. These include the increase in stage four sleep in the moderate light group as well as the reduction of SWS from sleep period 7 to 8 in the participants in whom melatonin was strongly suppressed. These findings are surprising because the effects of circadian parameters on SWS are thought to be minor [41].

Overall, the sleep and waking performance data corroborate the notion that the protocol and the bright light treatment affected the circadian system in a coherent way, i.e. shifted or reduced the amplitude of all aspects of the endogenous day-night cycle.

### Reduction in amplitude of overt circadian rhyhtmicity in multiple variables: Interpretation

The protocol led to changes in melatonin amplitude which were accompanied by changes in the amplitude of the rhythms in alertness, performance, core body temperature and cortisol, suggesting that broad aspects of circadian organization were affected. These observations highlight the disruptive effects of altered sleep-wake timing and light exposure on the temporal organization of physiology and behavior. Such disruptive effects are thought to be associated with negative health outcomes. Thus melatonin suppression is thought to mediate some of the negative effects of shiftwork on health [50]. The question remains how to interpret these effects on circadian amplitude within current conceptual frameworks of circadian organization. Traditionally a distinction is made between endogenous and exogenous components of overt rhythmicity. The suppression of the amplitude of multiple rhythms observed in the present protocol could conceivably reflect the direct effect of the continuous ∼90–150 lux moderate light exposure during the assessment on Day 9. That level of illuminance has previously been shown to exert a direct suppressive effect on melatonin [16],[14], [51] and influence alertness [17] although for cortisol effects of light have previously only been investigated for much higher illuminances [52] . It should be noted though that human circadian rhythmicity is in general considered to be robust under these constant conditions.

The extent to which light influences melatonin suppression differs between individuals [14] and depends on the light exposure history [18], [20], [53]. In a prior study conducted in our laboratory, we found that exposure to a 200 Lux light stimulus had more of a suppressive effect when participants were previously exposed to 2 lux, compared to when they were previously exposed to 200 lux [53]. The latter conditions is more comparable to the present study, because during the days preceding the melatonin assessment on Day 9, participants in the present study were exposed to light of the same (∼90–150 lux) illuminance.

An alternative interpretation of the observed reduction of circadian amplitude is that this reflects a change in the amplitude of the circadian system in response to the perturbation of entrainment. According to this interpretation, the shifted sleep-wake cycle and pattern of light and darkness prior to the assessment on Day 9 may have led to a reduction of the amplitude of the circadian pacemaker. During the sleep/wake schedule inversion, even the moderate light participants were exposed to light at times when the circadian system is most sensitive to light. It is therefore possible that the exposure to ambient moderate light exerted a sufficiently strong drive onto the pacemaker to suppress the amplitude of the circadian pacemaker itself in some of the participants. If so, this would be comparable to the amplitude reduction of core body temperature [10] and plasma melatonin [54] rhythms that we observed in some participants after exposure to critically-timed bright light.

As discussed elsewhere, such a reduction in amplitude may either reflect desynchrony of the individual neuronal oscillators which comprise the master pacemaker, desynchronisation between the central and peripheral oscillators, or a reduction of the amplitude of synchronized individual oscillators at the molecular level [55]. Of interest is that in humans light has been reported to directly induce the expression of PER2 in leukocytes [56], and a reduction of the amplitude of canonical clock genes has been observed after exposure to light in the Siberian hamster [57].

Whatever interpretation is correct, our findings demonstrate large inter-individual differences in the disruptive effects of the protocol on circadian organization. In real life, people are exposed to altered light dark and sleep-wake cycles during shift-work and time-zone travel. The present data and other recent data [14], [51] suggest that such schedules together with the associated light exposure can substantially reduce the amplitude of multiple circadian rhythms, and that individuals differ in their response. Whether and how this relates to individual differences in complaints and negative health outcomes associated with jet lag and shift-work remains to be established.

## Supporting Information

Figure S1 **Average waveform of performance during a constant routine in participants with a reduction in melatonin amplitude >50% (closed symbols) compared to those in whom the reduction was <50% (open symbols).** All data are aligned with the timing of the fitted minimum of the core body temperature rhythm. Error bars indicate 1 SEM.
(TIF)

Table S1 **Individual values of the change in melatonin amplitude as well as the timing of the melatonin midpoint.** See main text for detail. Time is in decimal hours, 28.5 = 04:30AM.
(DOCX)
