# Supplementary figures and images for: Amplitude Reduction and Phase Shifts of Melatonin, Cortisol and Other Circadian Rhythms after a Gradual Advance of Sleep and Light Exposure in Humans
Source: PLoS One. 2012 Feb 17;7(2):e30037. doi: 10.1371/journal.pone.0030037 (PMC3281823; doi:10.1371/journal.pone.0030037)

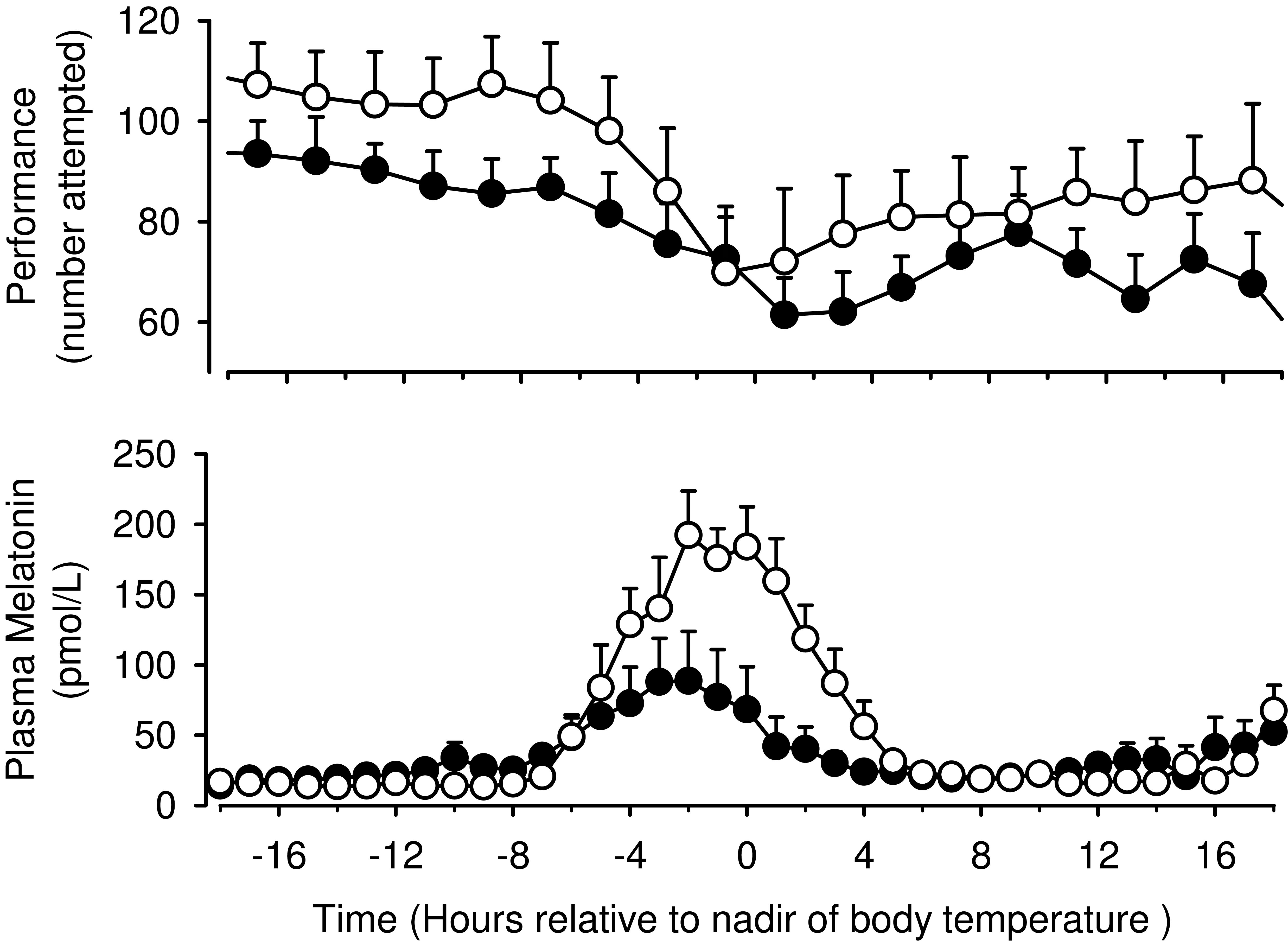

Supplement: Figure S1 — Average waveform of performance during a constant routine in participants with a reduction in melatonin amplitude >50% (closed symbols) compared to those in whom the reduction was <50% (open symbols). All data are aligned with the timing of the fitted minimum of the core body temperature rhythm. Error bars indicate 1 SEM. (TIF) [file pone.0030037.s001.tif]
