# Supplementary material for: Amplitude Reduction and Phase Shifts of Melatonin, Cortisol and Other Circadian Rhythms after a Gradual Advance of Sleep and Light Exposure in Humans
Source: PLoS One. 2012 Feb 17;7(2):e30037. doi: 10.1371/journal.pone.0030037 (PMC3281823; doi:10.1371/journal.pone.0030037)
Supplement: Table S1 — Individual values of the change in melatonin amplitude as well as the timing of the melatonin midpoint. See main text for detail. Time is in decimal hours, 28.5 = 04:30AM. (DOCX) [file pone.0030037.s002.docx]

| Participant  Moderate Light | Change Melatonin Amplitude (%) | Melatonin  Midpoint  Baseline  (Dec Hour) | Melatonin  Midpoint  CR | Participant  Bright Light | Change Melatonin Amplitude (%) | Melatonin Midpoint  Baseline | Melatonin Midpoint  CR |
| --- | --- | --- | --- | --- | --- | --- | --- |
| 1025v1t2 | -93.7844 | 28.5 | NA | 1041v | -22.1361 | 27.92 | 19.36 |
| 1028v1t2 | -37.5655 | 27.44 | 25.77 | 1101v | -58.1841 | 28.87 | 18.5 |
| 1048v | -63.5492 | 28.12 | 30.68 | 1102v | -84.2725 | 27.81 | 13.8 |
| 1060v | -42.9791 | 29.05 | 27.42 | 1110v | -12.3213 | 28.00 | 18.28 |
| 1141v | -96.3500 | 29.14 | NA | 1134v2t2 | -79.1211 | 27.69 | 19.35 |
| 1231v | -24.3526 | 26.37 | 24.5 | 1230v | -39.3900 | 27.97 | 21.26 |
| 1262v | -16.6926 | 28.81 | 26.75 | 1263v | -66.2969 | 28.33 | 18.44 |

Table S1. Individual values of the change in melatonin amplitude as well as the timing of the melatonin midpoint. See main text for detail. Time is in decimal hours, 28.5= 04:30AM
